# Direct Targeting of CXCR2 Receptor Inhibits Neuroblastoma Growth: An In Vitro Assessment

**DOI:** 10.3390/ph18101547

**Published:** 2025-10-14

**Authors:** Rameswari Chilamakuri, Deepika Godugu, Saurabh Agarwal

**Affiliations:** Department of Pharmaceutical Sciences, College of Pharmacy and Health Sciences, St. John’s University, New York, NY 11439, USA

**Keywords:** SB225002, CXCR2, IL-8, neuroblastoma, pediatric cancer

## Abstract

**Background**: This study addresses an important vulnerability in the treatment of high-risk neuroblastoma (NB). NB is characterized by high rates of metastasis, drug resistance, relapse, and treatment-related toxicities. Current treatments, which include intensive chemotherapy, surgical removal of tumors, and stem cell transplants, have less than 50 percent survival rates among high-risk NB patients, demonstrating the need for novel targeted treatment approaches. CXC chemokine receptor 2 (CXCR2), a G-protein-coupled receptor, has been implicated in promoting cancer cell proliferation, invasion, metastasis, angiogenesis, chemoresistance, and maintaining cancer stem cells. **Methods**: We analyzed transcriptomic data from 1464 primary NB patient samples to evaluate the prognostic significance of CXCR2 expression. Pharmacological inhibition of CXCR2 using SB225002, a selective small-molecule antagonist, was evaluated to determine its effects on cell growth, colony formation, apoptosis, and cell cycle progression in different NB cell lines. Three-dimensional (3D) spheroid models were used to examine tumor growth under physiologically relevant conditions. Mechanistic studies included gene expression analyses and immunoblot validation of key signaling regulators. **Results**: High CXCR2 expression was found to be inversely correlated with overall survival in patient datasets, suggesting a role in NB pathogenesis. Treatment with SB225002 significantly inhibited NB proliferation and colony formation while inducing apoptosis and cell cycle arrest in a dose-dependent manner. In 3D spheroid models, SB225002 significantly impaired spheroid formation and growth, confirming its potent anti-tumor efficacy. Mechanistically, CXCR2 blockade inhibited the expression of key pathway targets, including *GLIPR1*, *BACH2*, *JUN*, *CHEK1*, *AKT1*, and *CXCR2* itself. Immunoblot analysis confirmed significant inhibition of CXCR2 and GLIPR1 protein levels in response to SB225002 treatment. **Conclusions**: Taken together, our findings demonstrate that pharmacological inhibition of CXCR2 using SB225002 effectively inhibits NB tumor cell growth and tumorigenicity by modulating oncogenic signaling networks. This study provides strong evidence for elucidating CXCR2-targeted therapies as an attractive treatment option for NB. These findings support the development of CXCR2-targeted therapies for high-risk NB.

## 1. Introduction

The development and progression of tumors are significantly influenced by chemokines and their receptors [1]. Chemokines are primarily secreted by immune cells but can also be secreted by non-immune cells, such as vascular endothelial cells. C-X-C chemokine receptor 2 (CXCR2) is a prominent G-protein-seven transmembrane-coupled receptor that binds to a plethora of chemokines such as CXCL1, CXCL2, CXCL3, CXCL5, CXCL6, CXCL7, and CXCL8 (IL-8) to trigger its action [1,2]. CXCL3 is the strongest agonist, and CXCL5 is the weakest against the CXCR2 receptor. CXCR1 and CXCR2 are also known as IL-8 RA and IL-8 RB receptors. CXCR1 shares approximately 77% sequence homology with CXCR2, but the differences at the end sequence play an important role in ligand binding specificity [3]. However, CXCR1 expression in primary NB tumors and cell lines is generally lower and more variable compared to CXCR2, which is consistently upregulated in NB and plays a dominant role in recruiting neutrophils and myeloid-derived suppressor cells (MDSCs) to the tumor microenvironment, promoting angiogenesis, inflammation, and tumor progression [4,5].

IL-8 binds to both CXCR1 and CXCR2. Activation of CXCR2 induces dissociation of the G-protein-coupled receptor and releases Gβγ subunits from the Gα subunit. Uncoupled Gβγ subunits activate PLC (phospholipase C) and promote calcium mobilization from the endoplasmic reticulum to the cytosol, which is crucial for neutrophil chemotaxis [1]. PLC further promotes the phosphorylation of p38 and activates the NF-κB pathway. Activation of CXCR2 induces rapid phosphorylation of the ERK1/2 and PI3K/AKT pathways [1].

Aberrant activation of CXCR2 has been reported in multiple cancers, including neuroblastoma (NB), melanoma, colon, breast, ovarian, pancreatic, and prostate cancers [6]. In glioblastoma, the vascular system maintains the cancer stem cells (CSCs) via IL-8/CXCR2 signaling [7]. Increased IL-8 expression not only upregulated the expression of the CXCR2 receptor but also enhanced the migration of CSCs [7]. In esophageal carcinoma and leukemia, high amounts of IL-8 enhanced cell proliferation by dramatically upregulating CXCR2 expression [8,9]. The stroma-derived CCL2-promoted proliferation and CXCL12-mediated invasion were both amplified in PTEN-deficient prostate cancer due to the abnormal activation of the IL-8/CXCR2 signaling pathway [10]. Similarly, IL-8 expression upregulated the proliferation of lung cancer cells via EGFR transactivation [8] and the GAB2-dependent autocrine pathway in ovarian cancer [9]. Gastric cancer metastases are driven by interactions dominated by CXCR2 between tumor cells and macrophages [2]. Therefore, antagonizing the CXCR2 receptor could inhibit the receptor activity, inhibit tumor growth, and increase the sensitivity to chemotherapy [1].

NB is an extracranial solid pediatric tumor that originates from neural crest cells of the parasympathetic nervous system during the early embryonic stage. NB is highly heterogeneous, and this is reflected in patient survival rates. Low- and intermediate-risk NB patients have a >95% overall survival rate, high-risk NB patients have a <50% overall survival rate, and high-risk NB patients have a <50% long-term survival rate [10]. CXCR2 signaling enhances the invasive capacity of NB cells co-cultured with tumor-associated macrophages [11,12], and single-cell transcriptomics revealed that the upregulation of CXCR2 expression promoted NB cell proliferation via myeloid-derived suppressor cells [13].

Various small-molecule inhibitors that target the IL-8/CXCR2 signaling pathway are currently in phase 1/2 clinical trials, including AZD5069 (metastatic castration-resistant prostate cancer; NCT03177187), SX-682 (pancreatic ductal adenocarcinoma; NCT04477343), and MGTA-145 (multiple myeloma; NCT04552743). In the present study, we repurposed a CXCR2 inhibitor, SB225002, in NB. SB225002 [*N*-(2-hydroxy-4-nitrophenyl)-*N*′-(2-bromophenyl) urea] is a potent specific non-peptide, small-molecule antagonist of CXCR2 receptor [14], which was first developed for the treatment of inflammatory diseases to regulate neutrophil migration [15]. The anti-cancer activity of SB225002 has been reported in various cancers, including breast [16], ovarian [17], nasopharyngeal [18], esophageal [19], clear-cell renal cell carcinoma (CCRCC) [20], intrahepatic cholangiocellular carcinoma [21], and pancreatic cancer with activated K-Ras [22]. SB225002 has been reported to inhibit microtubule polymerization, angiogenesis, and cell cycle progression, promote mitotic catastrophe, manage acute and chronic pain, and inhibit gamma-secretase [17,23]. In the present study, we aim to evaluate the potential effects of SB225002 on NB in both MYCN-amplified and non-amplified cell lines, which is particularly relevant for high-risk patients. Our data from cell proliferation assays indicate that SB225002 inhibits NB cell growth in both two-dimensional cell culture and three-dimensional spheroid tumor models in a dose-dependent manner by inhibiting cell cycle progression. Additionally, we noted a dose-dependent activation of apoptosis in NB cells and a suppression of CXCR2 pathway genes and proteins following SB225002 treatments. Overall, our results indicate that SB225002 significantly inhibits NB growth and point out a new focused therapeutic approach against NB by targeting the CXCR2 pathway.

## 2. Results

### 2.1. CXCR2 Expression Inversely Correlates with NB Patients’ Survival

**Figure 1:**
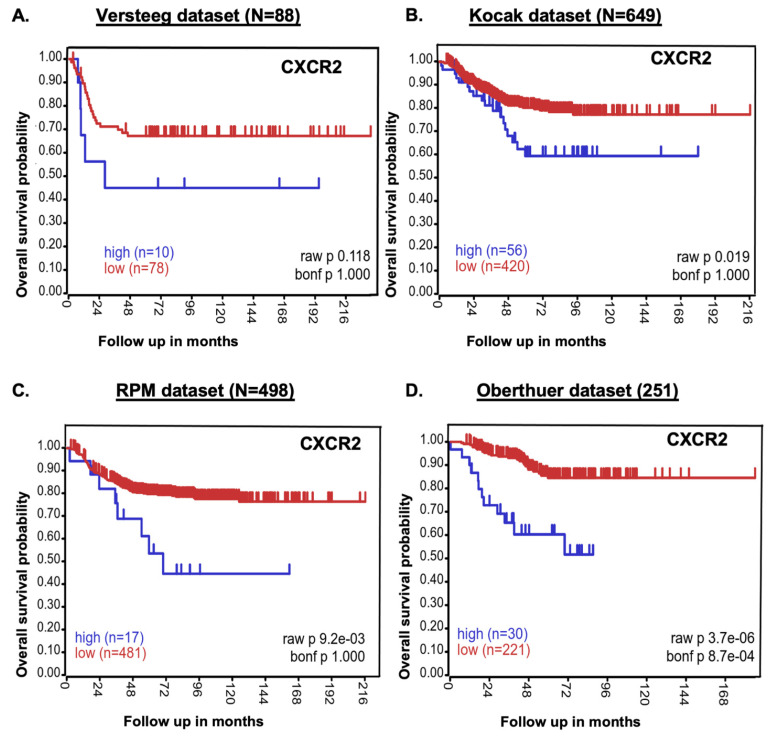
Poor NB patient survival is highly correlated with CXCR2 expression. The CXCR2 gene’s Kaplan–Meier survival analysis indicates a poor overall probability of survival for NB patients. (**A**) Versteeg, (**B**) Kocak, (**C**) RPM, and (**D**) Oberthuer datasets.

The role of CXCR2 in NB was examined using 1486 primary NB patient samples from four datasets to establish the link between the CXCR2 gene and overall NB patient outcomes. The Kaplan–Meier survival study showed that CXCR2 gene expression inversely correlates with NB patient survival. Low CXCR2 gene expression was associated with better overall survival in NB patients, while high expression was associated with poor prognosis (Versteeg dataset N = 88, *p* = 0.118; Kocak dataset N = 649, *p* = 0.019; RPM dataset N = 498, *p* = 9.2 × 10^−6^; Oberthuer dataset N = 251, *p* = 3.7 × 10^−6^; Figure 1).

Although the Versteeg dataset did not reach statistical significance, likely due to limited sample size, the consistent and significant associations observed in the three larger cohorts strongly support CXCR2 as a prognostic factor in NB. In contrast, analysis of patient datasets for CXCR1 (Supplementary Figure S1A–D) showed that high CXCR1 expression correlated with improved survival, suggesting it is not a driver of NB progression. Furthermore, CXCR1 transcripts were undetectable in NB cell lines compared to CXCR2 (Supplementary Figure S1E). Collectively, these findings highlight CXCR2 as a key prognostic marker and therapeutic target in NB.

### 2.2. SB225002 Inhibits NB Cell Proliferation

**Figure 2:**
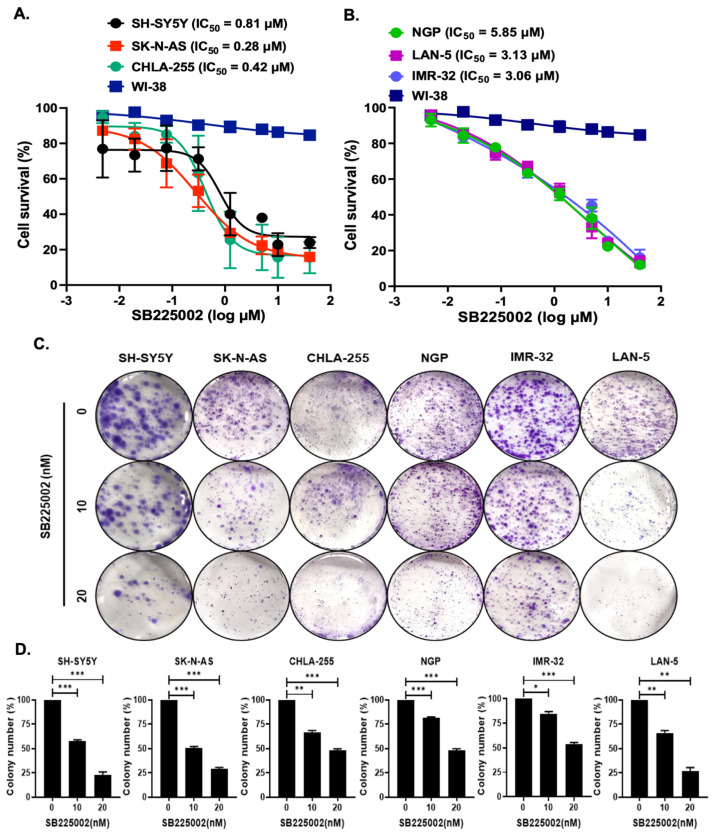
NB cell lines in response to different concentrations of SB225002. (**A**,**B**) Cell proliferation assays in (**A**) MYCN-non-amplified and (**B**) MYCN-amplified cell lines. (**C**,**D**) Colony formation assays, and their quantitative data represent relative inhibition of colony formation. * *p* < 0.05, ** *p* < 0.01, and *** *p* < 0.001.

Additionally, SB225002, a specific small-molecule inhibitor, was used to inhibit the CXCR2 receptor. Control non-cancerous fibroblast cell lines (WI-38) and six NB cell lines, including MYCN-amplified (NGP, LAN-5, IMR-32) and MYCN non-amplified (SH-SY5Y, CHLA-255, SK-N-AS), were used in cell proliferation experiments. In comparison to control fibroblast cell lines, the results showed that SB225002 strongly inhibited NB cell proliferation in both MYCN-amplified and MYCN non-amplified cell lines (Figure 2). For SK-N-AS, the IC50 values are 0.28 µM, while for NGP, they are 5.85 µM (Figure 2A,B). Notably, MYCN-amplified cell lines exhibited higher IC50 values than non-amplified cell lines, which may be attributable to elevated CXCR2 expression.

Additionally, a colony formation assay was used to confirm the anti-proliferative effect of SB225002 in all six NB cell lines. The results demonstrated that SB225002 significantly inhibited NB colony growth compared to controls (Figure 2C,D). Overall, these findings demonstrated that SB225002 effectively suppresses NB cell proliferation when it targets CXC2.

### 2.3. SB225002 Induces Apoptosis and Blocks Cell Cycle Progression in NB

**Figure 3:**
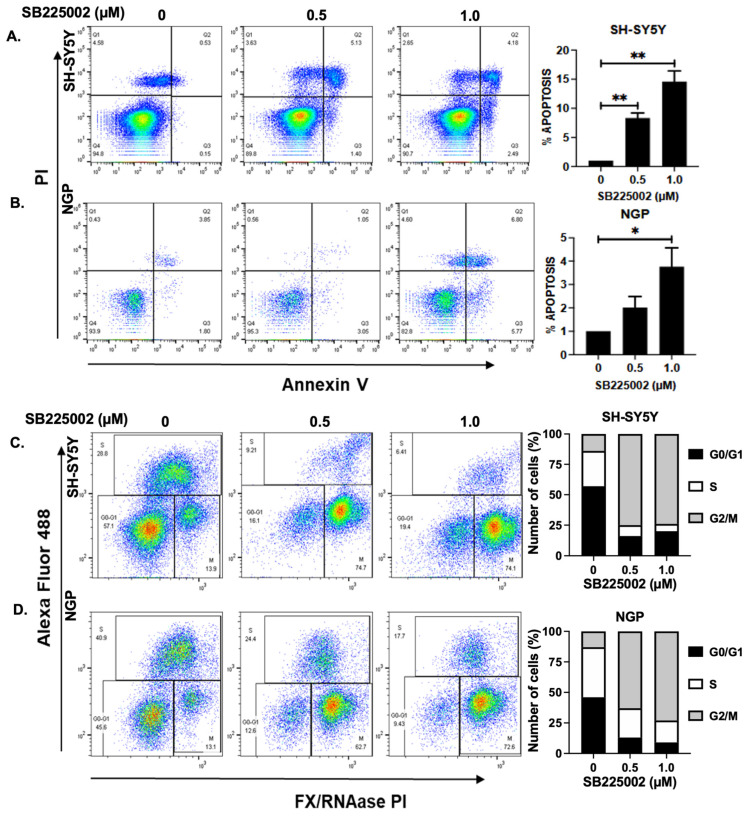
SB225002 induces apoptosis (**A**,**B**) and blocks NB cell cycle progression (**C**,**D**) in both MYCN non-amplified cell line SH-SY5Y and MYCN-amplified cell line NGP, in dose-dependent manner. * *p* < 0.05, ** *p* < 0.01.

To ascertain the effects of SB225002, we also conducted apoptosis and cell cycle studies in two NB cell lines, SH-SY5Y (MYCN-non-amplified) and NGP (MYCN-amplified). Our results show that SB225002 treatment significantly and dose-dependently increases the percentage of early apoptotic cells in NGP and SH-SY5Y by approximately 4 and 14 times, respectively, compared to control treatments (Figure 3A,B). Furthermore, SB225002 treatments significantly impede cell cycle progression, according to cell cycle studies conducted in NB cell lines. In SH-SY5Y and NGP cells, cell cycle S phase was shown to be approximately 4.5 and 2.3 times lower than the control, respectively, when CXCR2 was inhibited by SB225002 (Figure 3C,D) Additionally, we found that SB225002 blocked NB cell cycle progression at the G2/M phase, which was evident by about a 5.6-fold increase in the percentage of cells in the G2/M phase in both SH-SY5Y and NGP cells (Figure 3C,D). This data further confirmed the efficacy and cytotoxicity effects of SB225002 by inducing apoptosis and arresting NB cell cycle progression.

### 2.4. SB225002 Inhibits NB 3D Spheroid Growth

**Figure 4:**
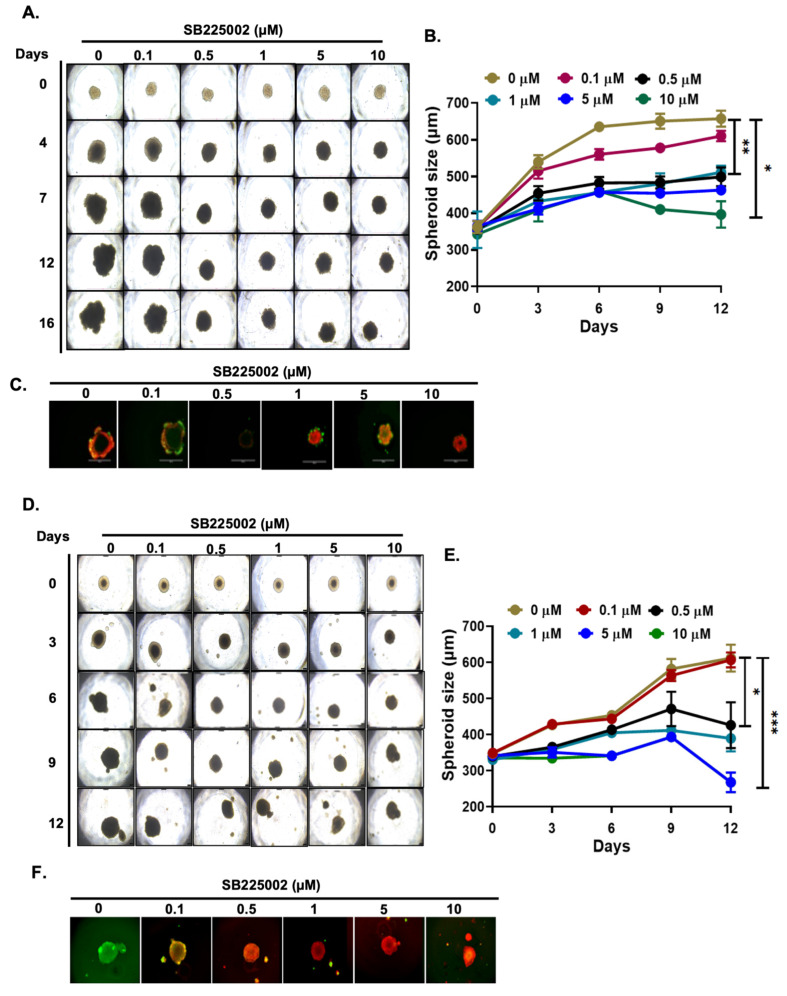
SB225002 inhibits NB 3D spheroid tumor growth in (**A**–**C**) SH-SY5Y and (**D**–**F**) NGP cells. (**C**,**F**) Calcein AM (green; live cells) and EthD-III (red; dead cells) fluorescence dyes were used to stain spheroids on the terminal day. Scale bar: (**A**,**C**,**D**,**F**) 750 μm. * *p* < 0.05, ** *p* < 0.01, and *** *p* < 0.001.

Further, we validated the anti-proliferative activity of SB225002 using two NB 3D spheroid tumor models developed using the SH-SY5Y and NGP cells (Figure 4). Three-dimensional spheroids closely recapitulate the in vivo tumor growth characteristics of solid tumors, including NB. The results from our 3D spheroid tumor studies demonstrate that SB225002 significantly, in a dose-dependent manner, inhibits spheroid tumor growth compared to control treatments in both cell lines (Figure 4A,B,D,E). Additionally, SB225002 significantly inhibits the live cells and increases the dead cells in spheroids, as determined using live (calcein-AM) and dead (EthD-III) cell staining of the terminal day spheroids (Figure 4C,F). Overall, the results of the 3D spheroid studies demonstrate that SB225002 significantly and dose-dependently inhibits NB spheroidal tumor development and growth.

### 2.5. SB225002 Inhibits the CXCR2 Receptor Pathway

**Figure 5:**
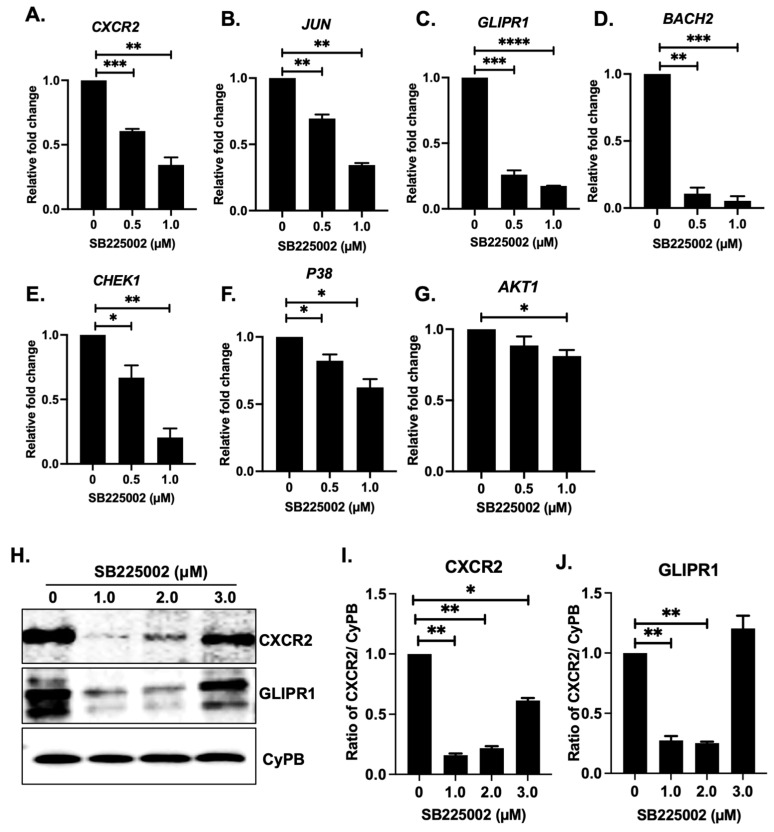
SB225002 antagonizes the CXCR2 receptor. (**A**–**G**) Gene expression analysis of CXCR2 receptor and downstream signaling pathways in response to SB225002. (**H**–**J**) Immuno-blot analysis of CXCR2 and GLIPR1 in response to SB225002 and corresponding densitometric analysis. * *p* < 0.05, ** *p* < 0.01, *** *p* < 0.001, and **** *p* < 0.0001.

SB225002 is a selective and potent inhibitor of CXCR2 and consequently affects downstream signaling through the CHK1 pathway. To delineate its molecular effects in NB, we performed gene expression and Western blot analyses. RT-qPCR demonstrated that SB225002 dose-dependently suppressed mRNA expression of *CXCR2*, *JUN*, *GLIPR1*, *BACH2*, *AKT1*, *CHEK1*, and *P38* genes compared with controls (Figure 5A–G). At the protein level, SB225002 significantly reduced CXCR2 and GLIPR1 expression levels. Interestingly, Western blot analysis revealed a biphasic response, in which CXCR2 and GLIPR1 levels initially decreased but subsequently increased at higher SB225002 concentrations (Figure 5H–J). This pattern may be due to the activation of stress-response pathways at elevated drug concentrations, altered protein stability, or the release of feedback inhibition of upstream regulators.

Collectively, these data demonstrate that SB225002 inhibits CXCR2 at both the transcriptional and protein levels, thereby blocking CXCR2-driven oncogenic signaling and suppressing NB growth.

## 3. Discussion

CXCR2 is ubiquitously expressed and is important for many different biological functions, including cell motility, cell adhesion, membrane trafficking, actin cytoskeleton reorganizations, and membrane receptor signaling [1]. The expression of CXCR2 is predominantly high in solid tumors, including NB, and is known to control the regulation of the PI3K/AKT, ERK1/2, NF-κB, and JAK/STAT pathways [24,25]. The CXCR2 chemokine signaling axis controls a range of tumorigenic processes, including cancer cell proliferation, invasion, metastases, self-renewal ability, and angiogenesis [2,26]. Inhibition of CXCR2 signaling suppressed the metastatic ability of colorectal, hepatocellular, and breast cancer cells [27,28]. Recent studies emphasize the wide-spectrum anti-tumor potential of SB225002 in multiple cancers by targeting CXCR2. SB225002 specifically decreases cell viability and induces apoptosis and cell cycle arrest (G2/M) in colorectal cancer in stromal cells [29]. In breast cancer, the IL-8/ CXCR2 axis inhibition by SB225002 also proves its high effectiveness in preventing tumor growth and metastasis.

Our findings, consistent with previous reports, demonstrate that SB225002, a selective CXCR2 antagonist, exerts potent anti-tumor activity in NB cell lines and 3D spheroid models, which more closely recapitulate in vivo tumor physiology [26]. SB225002 suppressed proliferation, induced apoptosis, and caused G2/M cell cycle arrest through disruption of key regulators, including CXCR2, PI3K/AKT, CHEK1, P38, and GLIPR1. These results are consistent with studies in colorectal, breast, and cervical cancers, where SB225002 similarly inhibited tumor growth and metastasis via blockade of the IL-8/CXCR2 signaling axis [26,29].

Importantly, the mechanisms of SB225002 may extend beyond CXCR2 inhibition. Recent evidence indicates that SB225002 interferes with microtubule polymerization, which may contribute to its anti-tumor effects and raises the possibility of off-target activity that could influence therapeutic specificity. Nevertheless, CXCR2-driven effects remain central, as CXCR2-knockout mouse models of prostate and renal cancers exhibit comparable tumor suppression [30,31,32]. Furthermore, senescence-induced pancreatic stellate cells secrete CXCL1, CXCL2, and CXCL3, and blockade of the CXCR2 axis suppresses pancreatic cancer cell proliferation [33]. Similarly, SB225002 inhibited cervical cancer cell proliferation and induced apoptosis in a dose-dependent manner by blocking CXCR2–CXCL1 interactions [34]. In addition, SB225002-mediated inhibition of neutrophil recruitment in the NB tumor microenvironment parallels CXCR2-specific effects observed in other tumor types, further reinforcing its targeted action.

SB225002 has been reported to downregulate cholesterol biosynthesis in ALL (acute lymphoblastic leukemia) cells [14]. Transcriptome network analysis exposed that SB225002 induces ALL cell death via the p53 pathway and arrests the ALL cell cycle by activating the GLIPR1 pathway [14]. Conversely, we noted a dose-dependent decrease in GLIPR1 expression upon treatment with SB225002 at both the protein and mRNA levels. We also identified that SB225002 impedes NB cell cycle growth at the G2/M phase by disrupting the PI3K/AKT pathway, the cell cycle regulator CHEK1, and P38. Oncogenic RAS-mediated cellular transformation results in elevated expression of all CXCR2 ligands, significantly contributing to tumorigenesis. Research indicates an interaction between IL-8 and VEGF signaling, which further upregulates BCL2 expression [35]. A combination of SB225002 and temozolomide downregulated the expression of the anti-apoptotic gene, *BCL2*, to induce apoptosis [36]. Our data show that SB225002 induced apoptosis in NB cell lines in a dose-dependent manner compared to controls by inhibiting multiple CXCR2 pathway genes. SB225002 has been reported to significantly inhibit the HOXC10-mediated colorectal cancer metastasis in combination with anti-PD-L1 therapy [37]. SB225002 increased the efficacy of anti-PD-1 therapy by inhibiting the accumulation of PMN-MDSCs in gastric cancer cells [38]. In contrast, Li et.al. indicated that gastric cancer cell lines with high CXCL17 expression are less sensitive to SB225002 [39]. SB225002 eradicated the formation of the pro-tumor immune microenvironment and blocked the recruitment of neutrophils and macrophages [40].

DNA damage response (DDR) is one of the most traditional anti-cancer mechanisms, with CHK1 playing a critical role in DNA replication stress and DDR [41]. Dysregulation of cell-cycle-regulatory genes, such as CHK1, has been reported in multiple cancers, including NB [42]. SB225002 inhibited the expression of CHK1 in a dose-dependent manner in ovarian cancer cells [43], and we observed similar results in NB. SB225002 reduced the invasion ability of prostate cancer cells by inhibiting the expression of BSP, OPN, and MMP-2 proteins [44]. In contrast, the CXCR2 axis facilitates integrin-dependent peritoneal metastasis of colon cancer cells [45]. The CXCR2 axis has been reported to induce angiogenesis in liver cancer mouse models via IL-17A [46]. Nevertheless, the CXCL5-CXCR2 signaling pathway has been reported to promote tumor metastasis by interacting with lymphatic endothelial cells and head-and-neck malignancies. The activation of p38 by epithelial–stromal communication of the CXCL1-CXCR2 interaction restored the proliferation of ovarian cancer cells [47]. Our data show that SB225002 significantly inhibited the expression of p38 in NB cells.

Recent reviews also suggest that CXCR2 is a central regulator of both cancer progression and inflammation, and CXCR2 antagonists such as SB225002 showed potential as therapeutic agents due to their capacity to prevent myeloid-derived suppressor cell (MDSC) recruitment and facilitate immune responses in prostate, hepatocellular, lung, and thyroid cancers in preclinical studies [48]. In the microenvironment of neuroblastoma, the CXCL2/CXCR2 axis enhances the invasiveness of the tumor via the macrophage-derived signal, justifying the rationale of targeting CXCR2 in NB [49]. Furthermore, in tumor-associated neutrophils (TANs), CXCR2 mediates recruitment and protumor polarization, as N2 and SB225002 can have anti-tumor activity by inhibiting N2 TAN accumulation and enhance radiotherapy in nasopharyngeal carcinoma and synergizing with cisplatin in lung carcinomas [50]. The CXCL2/CXCR2 axis also plays a role in immune evasion and resistance to therapy in different types of cancer, where SB225002 suppresses recruiting neutrophils and tumor growth under stress in hepatocellular carcinoma models [51]. Current understanding supports the therapeutic flexibility of SB2250002 in interfering with CXCR2-induced oncogenic multiple pathways.

In conclusion, various studies have confirmed that SB225002 exhibits anti-tumor activity by antagonizing IL-8 binding to CXCR2 receptors. Our findings demonstrate an inverse correlation between CXCR2 levels and the overall outcomes of NB patients. SB225002 substantially suppressed the proliferation of NB cells in a dose-dependent manner by inducing apoptosis and obstructing the progression of the NB cell cycle at the G2/M phase. Our research emphasizes that SB225002 may serve as a novel therapeutic strategy for NB by directly targeting the CXCR2 receptor. To further explore efficient therapeutic strategies for NB patients, we will integrate SB225002 with existing therapies in future endeavors.

## 4. Materials and Methods

### 4.1. Cell Culture and Patient Dataset

The following cell lines were investigated: human NB cell lines NGP, LAN-5, IMR-32, SH-SY5Y, SK-N-AS, and CHLA-255, as well as WI-38, a non-cancerous fibroblast control cell line, and all of them were cultured and passaged as previously outlined [25]. MedChem Express, Monmouth Junction, NJ, USA, provided SB225002. The control groups for this study were treated with DMSO unless otherwise declared.

### 4.2. Clinical Patient Dataset

We analyzed a total of 1486 primary NB patient samples from four different NB patient datasets using R2: Genomic Analysis and Visualization Platform (https://hgserver1.amc.nl/cgi-bin/r2/main.cgi, accessed on 23 September 2024). This platform is publicly available and supports multiparametric analysis of NB patients with microarray profiles and gene expression data.

### 4.3. Cell Proliferation and 3D Spheroid Assays

NB cell proliferation assays and colony formation assays in response to SB225002 treatments were performed as described previously [52,53]. Cell proliferation assays were performed using MTT cell proliferation dye (L11939; Alfa Aesar, Ward Hill, MA, USA). NB 3D spheroids for SH-SY5Y and NGP cell lines were developed as described previously [52]. Spheroids were randomized and treated with increasing concentrations of SB225002 for 12 days. Images were taken every third day to analyze the spheroid size using the Leica DMi1 microscope and LASX version 3.10 software suite tools (Leica Microsystems, Buffalo Grove, IL, USA).

### 4.4. Apoptosis and Cell Cycle Assay

An Annexin V-FITC Apoptosis Detection Kit was used to assess the apoptosis of NB cells after 24 h of treatment with SB225002 (ThermoFisher Scientific, Waltham, MA, USA), as described previously [54]. Similarly, NB cell cycle analysis in response to SB225002 treatment for 24 h was performed using a Click-iT Plus EdU Alexa Fluor 488 Flow Cytometry Assay Kit (ThermoFisher Scientific), according to manufacturer’s instructions and as described previously [54]. Flow cytometry data was analyzed using the Flow Jo software version 10 (BD Biosciences, Franklin Lakes, NJ, USA).

### 4.5. Gene Expression and Immunoblotting Assays

RT-qPCR was used to analyze gene expression in SH-SY5Y cells after 24 h of SB225002 treatment, as previously described [52,53,54]. SYBR Green dye and the Quant Studio 3 Real-Time PCR System (ThermoFisher Scientific) were utilized, with GAPDH as a housekeeping gene. The reactions were carried out in triplicate and repeated three times. The RT-qPCR primers used in this investigation are shown in Table 1.

Immuno-blotting analysis, as described previously [52], was performed on SH-SY5Y cells after treatment with SB225002 for 24 h. Clarity ECL Western substrate (Bio-Rad, Hercules, CA, USA) was used to develop and visualize the blots. The immunoblots were visualized and documented using the ChemiDoc XRS Plus system (Bio-Rad). Cyclophilin B was used as a loading control, and densitometric analysis was performed using the ImageJ software version 1.54g. Antibodies used in the present study were anti-CXCR2 (A2889) and anti-GLIPR1 (A16490), purchased from ABclonal Technology, Woburn, MA, USA, and anti-cyclophilin B (43603S) and anti-rabbit IgG HRP-linked secondary antibody (7074S) from Cell Signaling Technology (Danvers, MA, USA).

### 4.6. Statistical Analysis

Results are reported as mean ± standard error of replicates unless otherwise stated. Unless otherwise indicated, all experiments were conducted with a minimum of three biological replicates, and each measurement was derived from at least three technical replicates. Data was normalized and graphed using control treatment groups. GraphPad Prism 9 calculated IC50 values and statistically analyzed the data. The drug treatment groups’ statistical significance was determined by Student’s two-tailed *t*-test.

## 5. Conclusions

This study identifies SB225002 as a promising therapeutic candidate for NB, demonstrating efficacy in both conventional cell lines and 3D spheroid models that better recapitulate the in vivo tumor architecture. By selectively inhibiting CXCR2 signaling, SB225002 suppressed NB cell proliferation, induced apoptosis, and caused G2/M cell cycle arrest in a dose-dependent manner. These findings highlight the dual impact of SB225002 on intrinsic oncogenic signaling and extrinsic tumor-supportive processes, underscoring its potential as a stand-alone therapy or in rational combinations with other drugs. Future studies should focus on validating the effects of SB225002 in preclinical in vivo models, exploring pharmacological properties, and assessing synergy with targeted or immunomodulatory therapies. Overall, this work advances our understanding of CXCR2-targeted strategies and provides a strong rationale for the translational development of SB225002 to improve outcomes in NB and other CXCR2-driven malignancies.

## Figures and Tables

**Table 1:** RT-qPCR primers used in the study.

S.No.	Gene	Forward Primer (5′-3′)	Reverse Primer (5′-3′)
1	*CXCR2*	CTCCAATAACAGCAGGTCAC	GGCTCAGCAGGAATACCA
2	*JUN*	CCCCAAGATCCTGAAACAGA	CCGTTGCTGGACTGGATTAT
3	*GLIPR1*	AGCTGCACCCAAACTTCACT	ATCTGCCCAAACAACCTGAG
4	*BACH2*	GAAAACGATGCTGCCATTTT	TTGGTGCACACTTCTGCTTC
5	*CHEK1*	GACTGGGACTTGGTGCAAAC	TGCCATGAGTTGATGGAAGA
6	*P38*	CGCAAGGTCACTGGAGGAAT	CTGGGCTTTAGGTCCCTGTG
7	*AKT1*	GCACAAACGAGGGGAGTACA	AAGGTGCGTTCGATGACAGT
8	*GAPDH*	CACCATCTTCCAGGAGCGAG	TGATGACCCTTTTGGCTCCC

## Data Availability

Data is contained within the article.

## Supplementary Materials

The following supporting information can be downloaded at: https://www.mdpi.com/article/10.3390/ph18101547/s1, Figure S1: Low *CXCR1* expression correlates with poor survival in NB patients.
