# Supplementary material for: Direct Targeting of CXCR2 Receptor Inhibits Neuroblastoma Growth: An In Vitro Assessment
Source: Pharmaceuticals (Basel). 2025 Oct 14;18(10):1547. doi: 10.3390/ph18101547 (PMC12566611; doi:10.3390/ph18101547)
Supplement: Supplementary file 1 [file pharmaceuticals-18-01547-s001.zip › pharmaceuticals-3885780-supplementary.pptx]

## Slide 1
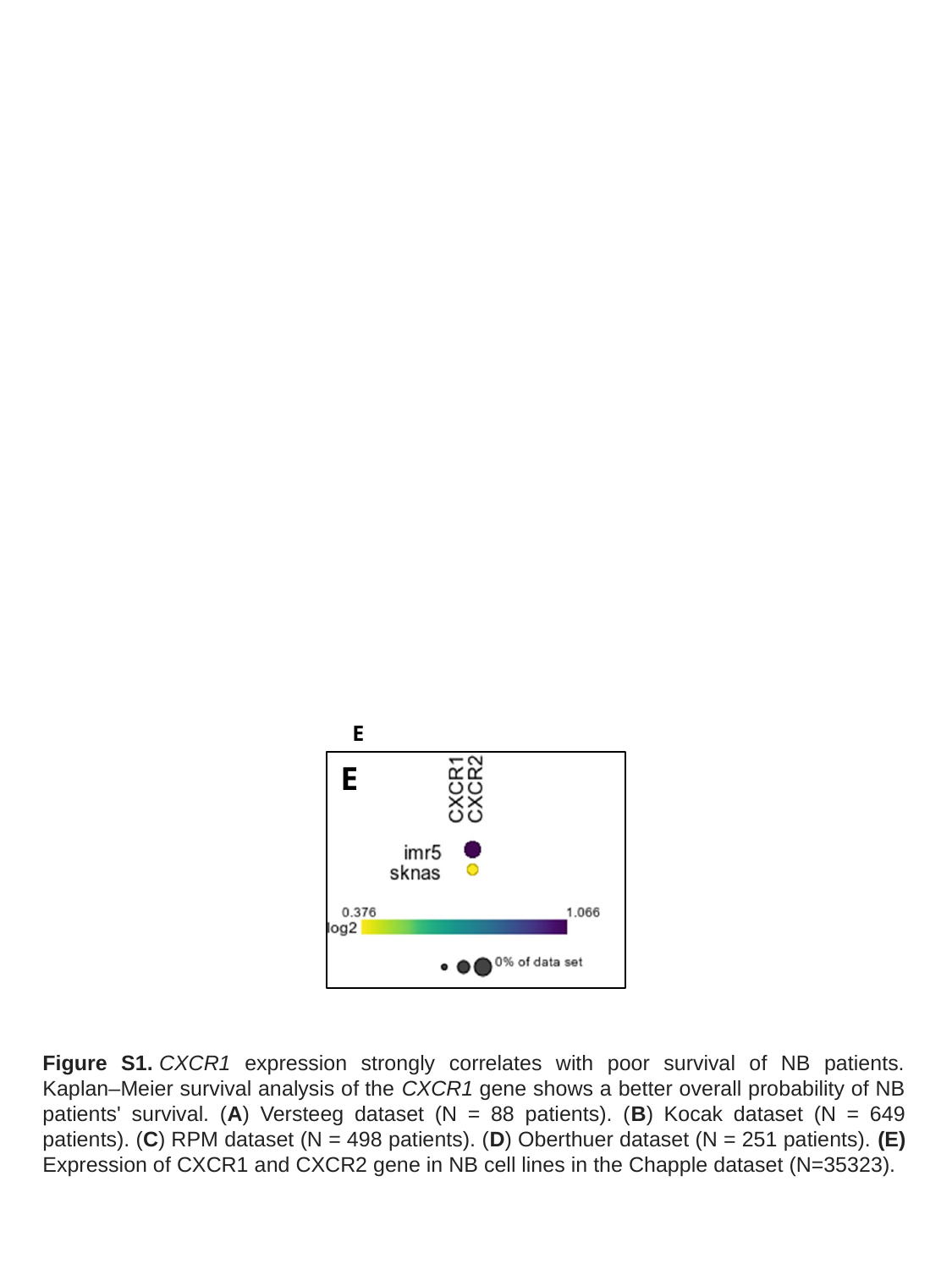

E
E
Figure S1. CXCR1 expression strongly correlates with poor survival of NB patients. Kaplan–Meier survival analysis of the CXCR1 gene shows a better overall probability of NB patients' survival. (A) Versteeg dataset (N = 88 patients). (B) Kocak dataset (N = 649 patients). (C) RPM dataset (N = 498 patients). (D) Oberthuer dataset (N = 251 patients). (E) Expression of CXCR1 and CXCR2 gene in NB cell lines in the Chapple dataset (N=35323).
